# Culturally-attuned AI: Implicit learning of altruistic cultural values through inverse reinforcement learning

**DOI:** 10.1371/journal.pone.0337914

**Published:** 2025-12-09

**Authors:** Nigini Oliveira, Jasmine Li, Koosha Khalvati, Rodolfo Cortes Barragan, Katharina Reinecke, Andrew N. Meltzoff, Rajesh P. N. Rao

**Affiliations:** 1 Paul G. Allen School of Computer Science and Engineering, University of Washington, Seattle, Washington, United States of America; 2 Institute for Learning & Brain Sciences, University of Washington, Seattle, Washington, United States of America; 3 Department of Psychology, San Diego State University, San Diego, California, United States of America; Mediterranean University of Reggio Calabria: Universita degli Studi Mediterranea di Reggio Calabria, ITALY

## Abstract

Constructing a universal moral code for artificial intelligence (AI) is challenging because human cultures have different values, norms, and social practices. We therefore argue that AI systems should adapt to culture based on observation: Just as a child raised in a particular culture learns the specific values, norms, and behaviors of that culture, we propose that an AI system operating in a particular human community could similarly learn them as well. How AI systems might accomplish this from observing and interacting with humans has remained an open question. Here, we propose using inverse reinforcement learning (IRL) as a method for AI agents to acquire culturally relevant values *implicitly* from humans. We test our approach using an experimental paradigm in which AI agents use IRL to learn different reward functions, which govern the agents’ actions, by learning from variations in the altruistic behavior of human subjects from two cultural groups in an online game requiring real-time decision making. We show that an AI agent learning from a particular human cultural group can acquire the altruistic characteristics reflective of that group’s average behavior, and can generalize to new scenarios requiring altruistic judgments. Our results provide a proof-of-concept demonstration that AI agents can be endowed with the ability to learn culturally-typical behaviors and values directly from observing human behavior.

## 1 Introduction

A formidable challenge in the field of artificial intelligence (AI) is endowing AI agents with the richness and depth of values and behaviors that characterize human cultures and their decision-making tendencies. State-of-the-art AI systems today [1–3] are based on large-language models (LLMs), which are neural networks with billions of parameters trained on data harvested from across the internet, leading to a “one-size-fits-all” (rather than pluralistic) AI system [4,5] (see also [6,7]). In this article, we propose that rather than attempting to handcraft or learn a universal code of values and behaviors for AI, AI agents should implicitly learn them by being embedded in the human culture in which they are operating [8]. We use the term “culture” here to refer to the dynamic values, norms, and behaviors shared by a social group [9], acknowledging that it is a dynamic phenomenon—a cultural cycle in which humans continuously shape and adopt values and behaviors [10,11]. The approach we propose would endow AI agents with the ability to deal with such cultural dynamics in a manner that is tailored to the culture or social group in which the AI agent was “raised.” This recognizes the fact that what is acceptable or normative in one culture may not be acceptable in another [12], necessitating AI systems that are capable of adapting to the values and behavioral tendencies of the culture in which they have been deployed. This will be challenging for AI and some intellectual humility is demanded, in as much as even humans, the creators of the AI, have a hard time appreciating and accepting the values and behaviors of cultures different from their own [12,13]. Might future AI surpass humans in such flexibility?

### 1.1 Altruism as a cultural value for AI to learn

In this article, we focus on the human tendency to act in a way that benefits others, even if it comes at a cost to oneself, often labeled “altruistic behavior.” For example, a person might give precious resources (such as food, money, or time) to someone else without directly benefiting themselves. In cases of extreme altruism, these acts can be directed towards strangers even in situations when the actor has no expectancy for reciprocal action or reward. There are significant between-country differences in altruistic tendencies across industrialized nations [14], small-scale traditional cultures [15], and even in children as a function of cultural background [16], motivating the need to investigate whether an AI system could learn altruistic behaviors from observation.

Our approach draws on evidence about how neural mechanisms in the brain support normative social behavior: the same reward systems thought to be involved in reinforcement-based learning are also harnessed to learn the dominant norms and values within a society [17–19]. The resulting system governs human cultural expectations and ways of interacting with others [20,21]. To learn what the accepted behavior is in any given context, the brain must first develop expectation rules about events and learn internal “value systems,” including the benefits of certain behaviors as well as the costs of deviating from the norm. For example, by perceiving or producing altruistic behaviors within a culture—such as giving others items of value or otherwise acting to benefit others even at a cost to oneself—a child or an adolescent may learn to connect, in a certain context, a “positive” value with behaving altruistically [16,22].

There has been considerable AI research on using inverse reinforcement learning (IRL) and imitation learning to mimic human actions [23–29]. More recently, IRL has been used to learn human value systems in specific contexts such as route choice modeling [30], and multi-objective IRL/RL methods have been proposed for human value alignment [31–34]. Here, we use IRL to address the question: *Can an AI agent learn cultural values and behavioral tendencies for specific social scenarios by observing the behavior of humans from different cultural groups, and then generalize this learning to a new context?*

### 1.2 Using inverse reinforcement learning (IRL) to train AI on human culture

IRL is a technique developed in the field of machine learning [23,26,28] to enable an AI agent to learn a new skill from human demonstrations; rather than simply mimicking the exact actions of the human, IRL attempts to learn a “reward function” from the human demonstrations. The reward function assigns positive or negative values (“rewards” or “penalties”) to particular features of the observed demonstration. By learning an appropriate reward function that is aligned with the presumed reward function being used by the human in the demonstrations, the AI agent is able to imitate the human behavior and generalize beyond simple mimicry of human actions.

In this article, we show how IRL can be used to train AI agents that can infer and learn the reward functions of humans belonging to a particular cultural group. The learned reward function assigns specific reward values (positive or negative) to situations in which the AI agent may find itself. By choosing actions that optimize total expected reward based on its learned reward function, the AI agent can behave in a manner that is attuned to the human culture in which it is embedded. A major advantage of this approach is that the agent can assign reward values to scenarios that are related to but not exactly the same as previously encountered scenarios. This allows the agent to generalize its decision-making ability, including those involving societal values and behavioral tendencies, to novel situations, emulating the first- and second-order generalization children demonstrate based on cultural learning and experience [16,35,36].

To test this approach, we collected data from human participants playing an online game involving simple forms of altruism and fairness, both of which have been regarded as important components of human morality and culture [37–42]. We recruited participants with cultural backgrounds associated with different altruistic behaviors [43], specifically, US participants who identified as White or as Latino (we use the term “Latino” to denote anyone born in or with ancestors from Latin America, regardless of gender).

Our hypothesis regarding differences in altruistic behavior is motivated by prior work showing that, within the same nation-state (here, the US), Latinos tend to be more collectivistic [44,45] and tend to have a more interdependent self-concept than Whites, who are generally characterized as being more individualistic and prone to seeing themselves as independent of others [46–48]. Whereas individualistic cultures tend to emphasize personal achievement and autonomy, collectivist cultures often prioritize group harmony and the achievements and welfare of others over personal gains. We hypothesize that, within the novel context of our online “world,” Latinos will exhibit more altruistic behavior than Whites. That is, subgroups of people within the US share cultural tendencies that they may bring to an online game, which we hypothesize can be inferred through IRL by an AI agent.

Note that rather than using data from human adults who make explicit value judgments, as in the case of the Moral Machines experiment involving the ethics of self-driving cars [49] or the trolley problem [50,51] (see also [52]), or data from descriptive judgments of scenarios presenting values and norms, as in the Delphi experiment [53] (see also [4,5]), our approach seeks to emulate the way a human child absorbs values and norms *implicitly* from observation and interactions with people in their culture [16,54]. Our approach shares similarities with previous work in reinforcement learning [55,56] and brain-inspired affective learning [57] showing how an AI agent could learn ethical characteristics such as altruism, empathy, and sympathy; these approaches did not train their AI agent on human data (as in our study here) and did not focus on cultural differences. Finally, our online game scenario includes an element of fairness, in addition to altruism: the “privileged” player has to endure some costs to help the other player who has been placed in an unfair and potentially less rewarding circumstance. We hypothesize that the reward functions inferred by IRL from human data may implicitly capture this aspect of the game scenario.

To summarize, this article tests the following hypotheses:

- H1: Cultural background and exposure to altruistic behavior may influence participants’ tendencies to display altruistic behavior.
- H2: An AI agent using inverse reinforcement learning can learn culturally attuned, quantifiable altruistic behaviors and values for specific social scenarios by observing the behavior of humans from different cultural groups.
- H3: An AI agent can generalize learned social values and behavior to a new context.

The remainder of the article focuses on the above hypothesis and presents our results. We first describe our experimental studies with humans and present results showing that an AI agent can learn from observing the behavior of a particular cultural group (Latinos or Whites) an IRL reward function capturing the altruistic tendencies of that group. We then show how this learning can generalize to new problems involving other altruistic decision making. Taken together, these results, though obtained from a simple online game scenario, point the way towards future AI agents that could become attuned to the culture in which they are operating by continually learning and adjusting their behavior from observing and interacting with humans within that culture.

## 2 Experimental studies with humans

**Fig 1:**
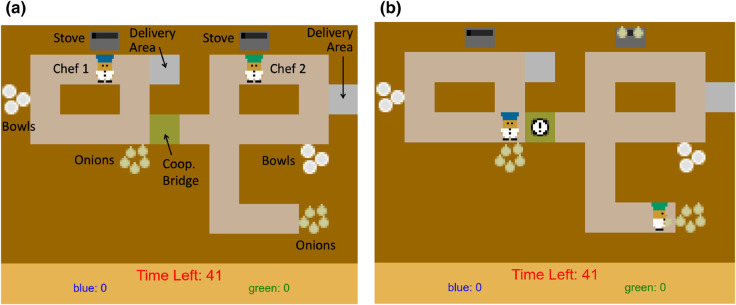
Testing altruistic behavior using an online experiment. **(a)** The screenshot shows both players, one in a blue chef’s cap on the left side (Chef 1) and the other in a green one on the right side (Chef 2), in front of their respective stoves (dark gray with a black bar inside). The goal is to cook and deliver as many onion soups as possible within a given time limit by putting three onions (yellow ovals with stalks) on the stove, picking up an empty bowl (white circle), collecting the cooked soup in the bowl from the stove, and delivering the bowl to customers (light grey location). Players have separate scores and can choose to cook soups individually (to get points) or share onions. Players can share onions by placing them on the “cooperation bridge” (square colored green between the two kitchens). Note that as depicted in (a), the path for the player on the left (blue cap) to get to the (continually replenished) store of onions (at the bottom of the kitchen) is much shorter compared to the path for the player on the right (green cap). Each study participant plays Round 1 (altruistic behavior baseline) on the left side, Round 2 (altruistic/non-altruistic bot demonstration round) on the right side, and Round 3 (behavior change examination round) on the left side again. When the computer-controlled bot is playing on the left in Round 2, it randomly selects one of two possible behaviors: altruistic behavior, where the bot places onions on the cooperation bridge for the human player, or non-altruistic behavior, where the bot does not help and focuses on maximizing its own score. **(b)** In this screenshot, the players are in front of their onion stores. The exclamation icon on the cooperation bridge denotes a call for help issued by the player on the right before going all the way to the southeast end to get onions from their own store.

We trained our AI on human behavior in an online experiment that required subjects to make altruistic decisions as part of a larger context of actions (details of the online data collection procedure are in the following section). Fig 1 presents a snapshot from the online experiment. The experiment used a version of the commercially successful “Overcooked” game in which players control chefs in a kitchen to prepare meals, given specific orders, within a time limit. We implemented a simplified version of the game, building on previous research in AI that used this game to study human-AI coordination [58].

As shown in Fig 1a, the online game involves two chefs (wearing blue and green hats), restricted to the left side and right side of the kitchen respectively, each controlled by either a human participant or the computer (see Methods for details on the online set up). The goal is to cook and deliver on your side of the kitchen as many onion soups as possible within a given time limit. This involves putting three onions in a pot on your stove, then picking up an empty bowl, putting the cooked soup in the bowl, and delivering the bowl of soup to a delivery area on your side of the kitchen (see annotations in Fig 1a). Before the game-related data collection began, our software taught participants how to play the game through a series of interactive tutorials. The participants also provided basic demographic information through the online software interface. In each of three rounds, the human participant was paired with another player on the other side of the kitchen (unbeknownst to the human participant, the other player was a computer-controlled bot). Cooking in one side of the kitchen (the right side) required more effort as the chef needed to traverse a longer path to obtain an onion and put it on the stove. This asymmetry created an unfair situation and the chef on the easier side (the left side) could alleviate this unfairness by passing an onion to the other chef through a “cooperation bridge” (green square in Fig 1a). Such an altruistic act, however, hurt the helping chef’s performance as it consumed time that could otherwise have been used to deliver more soup and therefore obtain more points.

In our experiment, human participants played three rounds: In Rounds 1 and 3, they controlled the chef on the left side of the kitchen (where the onions are closer to the stove). In Round 2, they controlled the chef on the right side (where the onions are farther away from the stove). When the computer-controlled bot was on the right side (i.e., rounds 1 and 3), it was programmed to call for help every time it moved empty-handed past the bridge connecting the two kitchens (indicated by an exclamation mark in Fig 1b). In Round 2, half of the participants were randomly assigned to be paired with a computer-controlled bot on the left side that demonstrated altruistic behavior by depositing an onion on the cooperation bridge for the human-controlled chef. The rest of the human participants were randomly paired with a self-serving bot that never shared the onions, resulting in the human player having to go to the more distant location to get onions. This design choice was meant to test the idea that participants who received help in Round 2 might increase their altruistic behavior in Round 3 compared to participants who did not. The overall goal remained the same: to achieve higher scores by cooking as many soups as possible in a 60-second round. The current scores for each player (blue and green chef) and the time left on the clock were always shown on the screen (see Fig 1). A chef’s score, whether human- or computer-controlled, increased by 10 points whenever the chef delivered a soup.

## 3 Results

We present three sets of results. The first set of results is from the online experiment investigating our hypothesis regarding behavioral differences across cultures pertaining to human altruistic decision-making. The second set of results shows how these differences in human altruistic behavior can be learned from human data using inverse reinforcement learning to create culturally-attuned AI agents capable of solving the Overcooked task. The third set of results illustrates generalization to different variations of this task, and a stronger form of generalization to a new task requiring altruistic decision making.

### 3.1 Cultural variations in human altruistic behavior in the online experiment

For the online experiment described above, we recruited 300 adult participants from two groups of US residents: one group self-identified as ‘White’ (*n* = 190) while the other self-identified as ‘Latino’ (*n* = 110). Data was collected across three major online recruitment platforms: Prolific, Positly, and Amazon MTurk. The dataset contained participants’ summary results per round and all game-playing information, i.e., sequential game-state snapshots, including each board arrangement, actions taken by both agents, time, and scores. Demographic information such as education level and political orientation was also collected (details in the Methods section).

**Fig 2:**
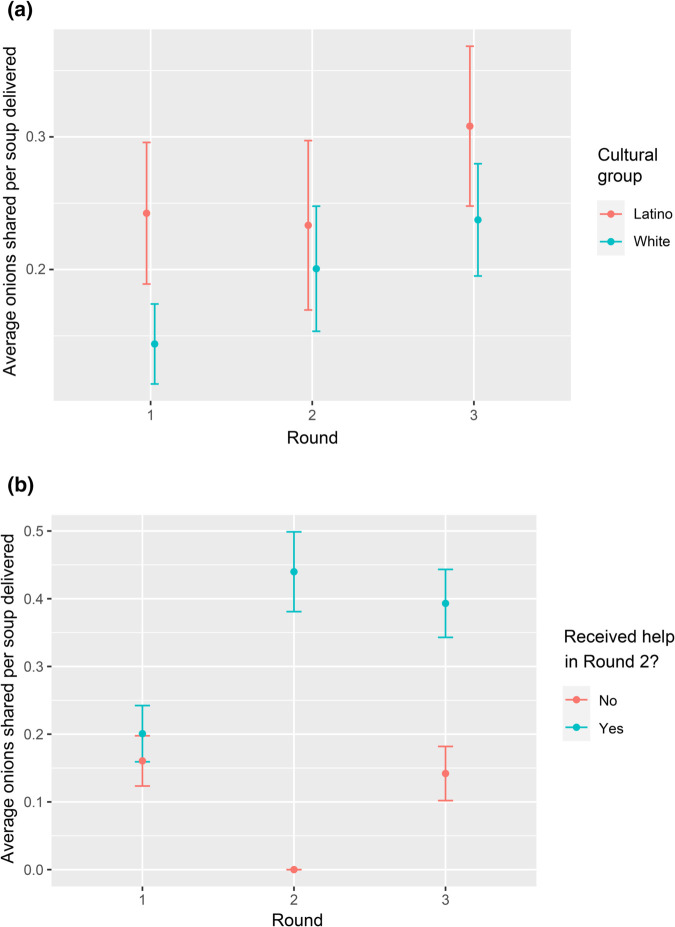
Human altruistic behavior in the online experiment. In both figures, the Y axis shows the average human altruistic behavior in terms of onions shared per delivered soup. **(a)** shows a tendency for Latinos to share more onions, although with statistical significance only in Round 1 (considering a confidence interval of 95%). **(b)** shows the effects of the computer-controlled bot helping versus not helping the human participant in Round 2 (see text for details). Participants who received help in Round 2 demonstrated significantly more altruistic behavior in Round 3 compared to Round 1. In contrast, those who were not helped maintained approximately the same level of altruistic behavior as Round 1. Note that the data points for Round 2 denote the average onions collected by the human player from the cooperation bridge per soup delivered.

We first tested our hypothesis, motivated by previous research on prosocial tendencies in Latino and White populations [45,47], that cultural heritage may influence participants’ tendencies to display altruistic behavior at the outset of the experiments. Fig 2a supports this hypothesis: Latino participants were more altruistic at the outset (in Round 1 of the online game) compared to White participants (ANOVA, *F* = 11.81 and *P*<0.001), with Latino participants averaging 0.24 (*SD* = 0.28) shared onions per delivered soup and White participants averaging 0.14 (*SD* = 0.21).

We next examined the hypothesis that participants who were randomly assigned to receive help in Round 2 may increase their helping behavior in Round 3 compared to those who did not receive help in Round 2. Fig 2b supports this hypothesis: ANOVA tests confirm a highly significant effect for those who received help (*F* = 26.89 and *P*<0.0001 – an increase of two times more shared onions per soup delivered) compared to those who did not receive help (*F* = 0.61 and *P* = 0.44). These results show that our study design captured differences in altruistic behavior (Cohen *d* = 0.28 when comparing Rounds 1 and 3). While the remainder of this article focuses on the fact that the two cultural groups in our dataset demonstrated different levels of altruistic behaviors in Round 1, we note that two other characteristics influencing participants’ behavior may be worth investigating in future studies: gender and political leaning (see S1 Appendix and S1 Fig in Supporting information).

### 3.2 Training AI agents with IRL to learn human cultural behaviors and values

We next tested our hypothesis that AI agents could learn cultural behaviors and values by observing and learning from the actions of humans from particular cultural groups. Specifically, we used inverse reinforcement learning (IRL) to recover the underlying reward functions for participants who identified themselves as Latino or White participants. We then tested (i) whether such an IRL-based AI relying on the learned reward function generates behavior aligned with the behavioral tendencies of the cultural group it was trained on, and (ii) whether the learned reward function allows the AI agent to generalize and make altruistic decisions when confronted with novel scenarios.

We quantified the altruistic tendency of IRL-based AI agents trained on a particular group’s Overcooked game data using the *Sharing Ratio (SR)* measure, calculated as the *average reward for the sharing trajectory (ART_S_*) divided by the *average reward for the cooking trajectory (ART_C_)* where the average reward of a trajectory (ART) is defined as:

$$ART(\tau ,rf)=[\sum_{t=1}^{n_{\tau }}norm(rf(t))]/n_{\tau }$$ (1)

where *τ* is a trajectory (for cooking or sharing behavior) with $n_{\tau }$ time steps, *norm* is the min-max normalization applied per trajectory step, and *rf* is the learned reward function for each of the four computed IRL models. The sharing and cooking trajectories were extracted from human participant traces as described in the Methods section (Model training section).

Defined as above, the SR for a given IRL model indicates how much more rewarding a group considers sharing an onion compared to using it for cooking their own soups: the higher the sharing ratio, the more altruistic the AI agent behaves.

**Fig 3:**
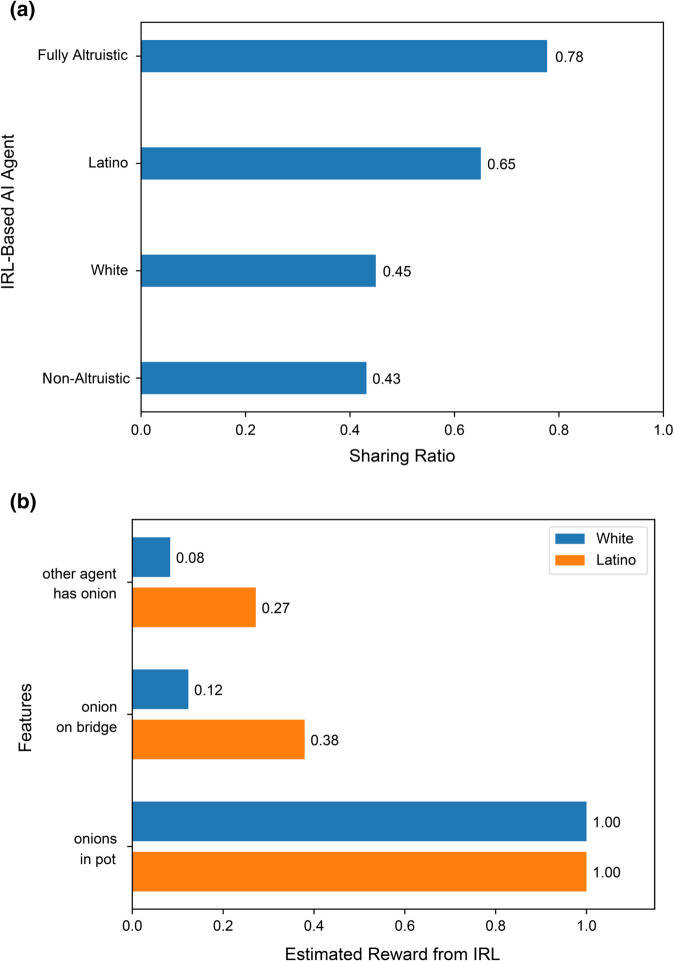
Inverse reinforcement learning (IRL) results based on human data from the online experiment. **(a)** The bars represent the Sharing Ratios computed from the behaviors (trajectories in the game) of the four different IRL-based AI agents on the Y axis (see text for details). **(b)** The scaled reward values assigned to three different features extracted from the game, showing how the selected features contribute to the final reward value estimated for White versus Latino participants.

Fig 3a compares the Sharing Ratio for the two IRL-based AI agents trained on the Latino and White participant groups, with two other agents trained on datasets for the two extreme behaviors, namely, a fully altruistic agent that shares every onion, and a non-altruistic agent that does not share any onions (see Model training in the Methods section). As expected, the fully altruistic agent and the non-altruistic agent had the highest and lowest sharing ratios, respectively. Note that these agents do not have sharing ratios at the two extremes (0 and 1) because the sharing and cooking trajectories share reward features such as “position relative to onion store” and “agent has onion” which have nonzero values for *ART*_*S*_ even for the non-altruistic agent, resulting in a sharing ratio >0 for this agent (and similarly for *ART*_*C*_ for the fully altruistic agent).

The sharing ratio for agents trained on Latino participants was closer to the fully altruistic agent’s sharing ratio and higher than the ratio for White participants, which in turn was higher than the ratio for the non-altruistic agent (Fig 3a). S2 and S3 Figs in Supporting Information (S2 Appendix) show example trajectories of the IRL-based AI agents trained on Latino and White participants.

These results indicate that the learned reward functions for the Latino and White participant groups are consistent with the altruistic tendencies for these groups reported in the previous section. They additionally help to quantify the extent of altruistic behavior of these groups in the game.

#### 3.2.1 Explainable AI

Besides estimating an intrinsic reward function for each cultural group in the game, the IRL models offer a unique opportunity to analyze how this knowledge is expressed in terms of different features of the task, in this case, features of the Overcooked game. This aspect of our approach to AI agents learning cultural values and behaviors fits well with the growing realization that deployed AI systems need to be explainable [59,60] rather than black-box models.

Fig 3b shows the reward values estimated using IRL for three of the most relevant features when comparing the Latino versus White cultural groups in our dataset. The fact that both groups highly valued having onions in the pot captures the explicit goal of the game (bottom two bars in the plot). Besides the common goal of cooking more soups, the reward function also captures the differences between the two cultural groups. The observation that the Latino participants provided more help to the other player is captured by another feature in Fig 3b: whether there is an onion on the cooperation bridge, which has a higher value for Latino participants than White participants. The third feature in Fig 3b – whether the other agent is holding an onion – is also directly related to a participant’s altruistic behavior. The learned reward value for this feature is higher for Latino participants than White participants, consistent with the overall differences in altruistic behavior for these two cultural groups seen in our data.

#### 3.2.2 First-order generalization to variations of the Overcooked game

**Fig 4:**
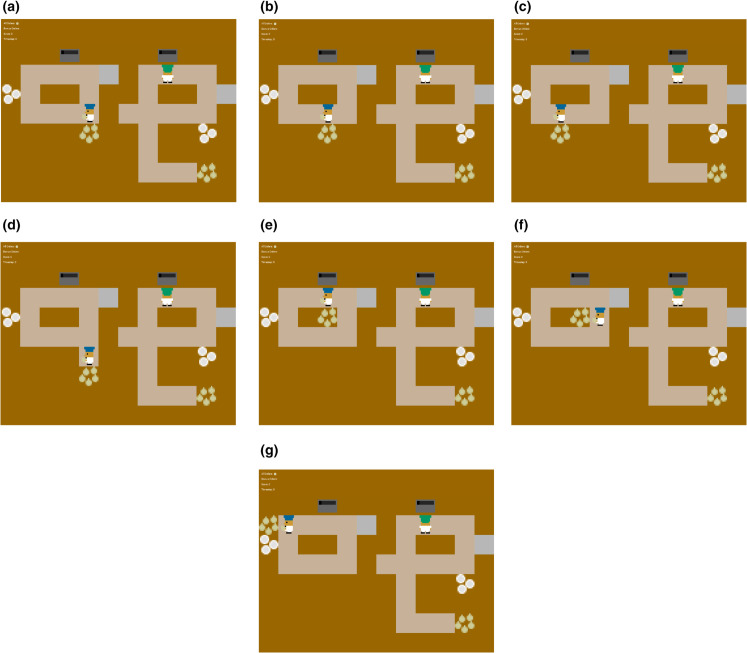
Six variations of the Overcooked task. By modifying the original layout shown in **(a)**, we created six new IRL environments by changing the player position, onion store position, and counter layout. The idea behind the changes was to alter the level of effort needed to share an onion. In the new layouts **(b)** through **(d)**, sharing an onion requires less steps (shorter distance) than to cook. Conversely, modified layouts **(e)** and **(g)** require more steps (longer distance) to render help than to cook, and modified layout **(f)** requires approximately the same number of steps for both helping and cooking. Collectively, these new layouts test the agent’s capacity for first-order generalization to new scenarios within the same game structure.

**Fig 5:**
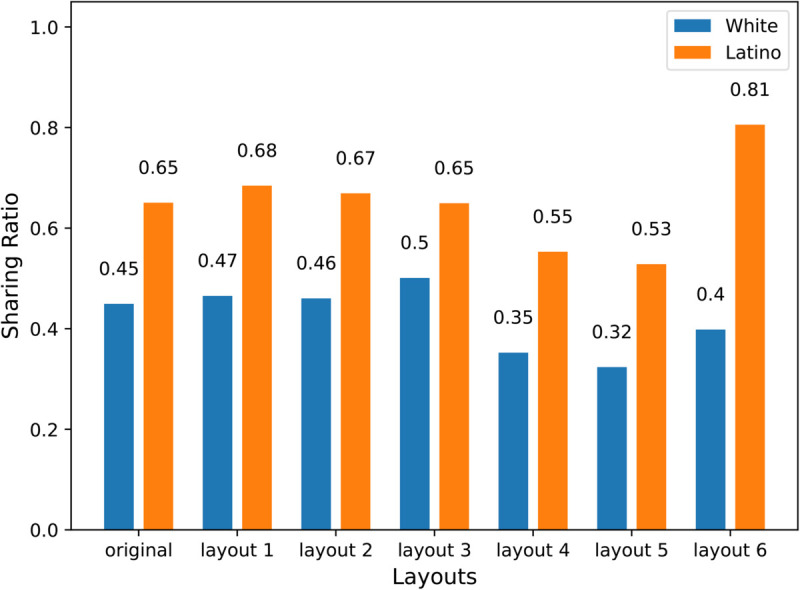
Performance of AI agents in six variations of the Overcooked task. Each bar represents the Sharing Ratio for an AI agent trained on the behavior of White or Latino participants. A higher ratio indicates a preference for helping the other player than cooking one’s own soup, based on the reward function learned from humans playing in the original game layout. The sharing ratio of the IRL agent trained on data from Latino participants was higher than the IRL agent trained on data from White participants in all layouts. In both the original layout and layouts 1 through 3, where it requires fewer steps to help than to cook, both IRL agents exhibit relatively high sharing ratios.

We examined whether the AI agent can make a first-order generalization to variations of the Overcooked task. Specifically, we used the reward function learned in the original game layout (Fig 1a) to test the agent’s performance for six other new spatial layouts with no further training. That is, we tested whether the reward function learned from the Latino versus White groups generalized to the different layouts and exhibited behavior congruent with the level of altruism shown by that group. The specific new layouts are shown in Fig 4. We summarize our findings in Fig 5. The results showed that an IRL-trained AI agent can use its learned reward function from the original layout to guide its behavior in the new layouts with no additional training. We call this form of generalization a ‘first-order’ generalization.

#### 3.2.3 Second-order generalization to a new context: The keep or donate problem

Next, we considered whether, under certain assumptions, an IRL-based AI model can exhibit something more powerful than first-order generalization. Specifically, can the AI agent use its “experience” from learning from humans to guide behavior in a novel task with few or no surface similarities with the original game? We call this second-order generalization, a more powerful form of generalization that involves applying previously learned reward functions to new problems that go beyond surface-level similarity to the original problem.

In our case, second-order generalization requires the agent to abstract beyond the features used in the reward function. Specifically, consider two of the features used for learning human behaviors in the Overcooked game: “other agent has onion” and “onions in pot” (Fig 3b). These two features are special cases of the more general features “other agent has the resource” and “I have the resource” respectively, where the resource can be onions, money, or any object important for the current task or scenario. We assume the agent can recognize this relationship between a specific resource (“onions”) and the general category of resources. This association was done by hand for the results in this section. Note that hand-crafted features have the potential to introduce bias which may hinder different types of generalization. Learning generalizable features from data across many tasks is an important direction for future research.

**Fig 6:**
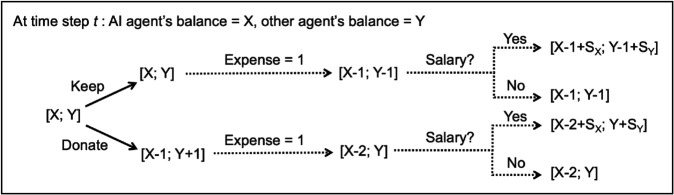
Generalization to a new scenario: The “Keep or Donate” problem. The diagram depicts the Markov dynamics of the “Keep or Donate” problem for a given time step when the expense for the time step is 1. After the expense is deducted, a salary of ${\text{S}}_{\text{X}}$ (for the AI agent) and ${\text{S}}_{\text{Y}}$ (for the other agent) is added if this time step was selected at random as a salary step (see text for details).

Here we illustrate how the generalized features (“other agent has the resource” and “I have the resource”) and the specific values for them learned from humans in the Overcooked game (Fig 3b, top and bottom bars) can potentially be used by the AI agent for altruistic decision-making in a different scenario. The new scenario, depicted in Fig 6, involves deciding whether or not to donate a portion of your current monetary savings (in arbitrary units) to another agent in need. Both the AI agent and the other agent have unpredictable expenses at each time step, resulting in a deduction of either 1 unit (with probability 0.8) or 2 units (with probability 0.2) from each agent’s current balance. Additionally, the AI agent gets a “salary” i.e., a periodic infusion of 5, 10 or 15 units every 6, 8 or 10 timesteps (chosen uniformly at random) while the other (resource-poor) agent gets a salary of 2 units at the same time step. The AI agent needs to decide, at each time step, whether to keep its current savings or donate 1 unit to the other agent. Each episode begins with the AI agent having 5 units and the other agent having 2 units, and lasts 100 time steps. The reward function includes the IRL-based reward feature values learned from humans in the Overcooked game (see table in Methods) and a penalty of –1 whenever the current balance goes to 0, capturing the undesirability of being left resourceless. Based on this reward function, Q-learning [61] was used to learn the policy for the AI agent as follows (see Methods for details): the state included X, the current balance of the AI agent, and Y, the current balance of the other agent; there were two possible actions: “Keep current balance” or “Donate 1 unit.” The Markov dynamics of the problem is illustrated in Fig 6.

**Fig 7:**
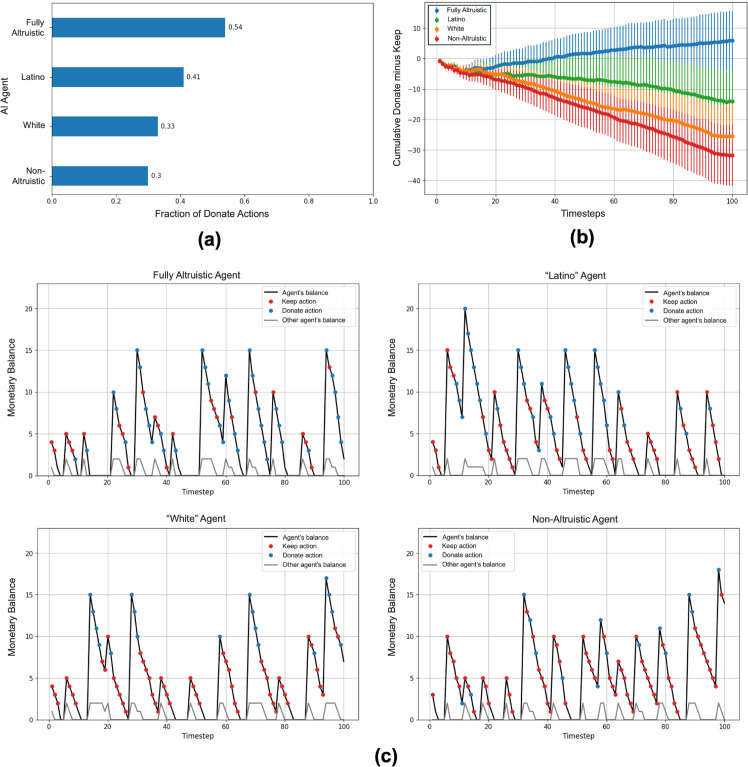
Results for the “Keep or Donate” problem. **(a)** Fraction of Donate actions for each type of agent, averaged over 100 episodes, each lasting 100 time steps. **(b)** Cumulative sum of Donate (+1) and Keep (–1) actions over the timecourse of an episode, averaged over 100 episodes (error bars show 1 standard deviation above/below mean). **(c)** Four example episodes showing the monetary balance and actions employed by each of the four trained AI agents.

Figs 7a-c show the results. Fig 7a shows the average fraction of Donate actions for each agent across 100 episodes. Overall, as seen in this plot, the “Latino” AI agent donated more often compared to the “White” AI agent (the values for altruistic and non-altruistic agents are also plotted for reference - see Methods for details). Fig 7b shows the evolution of altruistic/non-altruistic behavior for each agent over time: each plot shows the average of the sum of Donate (+1) and Keep (-1) actions from the first to the current time step (error bars show 1 standard deviation above/below mean). The four panels in Fig 7c provide examples of Donate versus Keep actions in a particular episode as time progresses. Besides the greater number of Donate actions for the “Latino” agent compared to the “White” agent, the agent behavior traces in Fig 7c illustrate how these agents tend to donate more when they have a higher balance and when the other agent’s balance has been depleted. We ran additional experiments to explore the effect of varying parameters such as the total number of time steps per episode and deduction probabilities governing expenses at each time step (see above). We found that the results above hold when these parameters are varied (see S3 Appendix in Supporting Information).

In summary, these results illustrate the utility of an IRL-based approach to learning culturally-attuned AI: assuming reward features can be generalized by the agent or by human design (as we did here), the IRL concept of learning reward functions rather than simply mimicking surface-level human actions can allow the AI agent to generalize the culturally-attuned values learned in a specific task (in this case, altruistic tendencies in the Overcooked game) to new scenarios that are different from the original task.

## 4 Discussion

Given the rapid strides being made in AI today, a question of utmost importance is how AI systems can be imbued with human values. We propose that AI agents could be culturally attuned and learn higher-level values and behaviors implicitly from observation and interactions with other humans within a social group. To illustrate this approach, we presented results from an online experiment involving 300 subjects. Our results suggest that inverse reinforcement learning (IRL) can be used by AI agents to learn culture-specific reward functions from the behavior of humans from a particular cultural background (here, US participants who self-identified as Latino or White). Specifically, an AI agent which learned its reward function from the behavior of Latino participants demonstrated a higher level of altruistic behavior reflecting what it had learned from this specific cultural group, even in variations of the original task as well as a novel scenario involving a different type of altruistic decision making.

Our results also show how the demonstration of altruistic behavior by another player in one round of interaction fosters altruistic behavior in human subjects in the next round, akin to behavioral accommodation in which people adjust their behavior to that of others. The degree of such behavioral accommodation was found to vary according to cultural background [62]. Our result regarding differences in altruistic behavior between Latino and White participant groups fits nicely with previous research reporting similar findings [43–45].

It is important to distinguish our approach to training AI agents from ones that use supervised learning to mimic the behavior of each group of participants, which would also yield an AI agent for each cultural group embodying the average behavior of that group (similar to the result we presented in Fig 3a). Our approach quantifies each cultural group’s reward system by mapping features of observed behaviors (Table 3) to quantitative positive/negative values (rewards) via inverse reinforcement learning (see Fig 3b). An important advantage of this approach is that the features-based reward function learned in one task can generalize to new situations where the same features may recur (Fig 5) and possibly in more abstract form in a new context (second-order generalization) (Figs 6 and 7). The ability to learn a function from features to reward values goes beyond simple mimicking of exact behavior and emulates a powerful human ability, namely, to generalize beyond observed behavior by inferring underlying “rewards” governing a human’s visible behavior and mapping such “rewards” to other situations. This ability could pave the way for AI learning adaptively from different human cultures, given sufficient examples of diverse human behaviors in different cultural groups. Such an approach helps alleviate the core social-scientific problem observed in the current “one-size-fits-all” approach to the training of AI, focusing on “foundation models” and LLMs.

Additionally, in contrast to other methods based on deep learning and LLMs, the proposed method based on a reward function learned by the AI agent from human behavior offers interpretability through analysis of the reward function’s features (Fig 3b), thereby contributing to explainable AI. Specifically, designers of AI systems could explicitly design the system to include reward function features that promote interpretability, similar to our features “other agent has onion” and “onions in pot.” Having interpretable features in an AI system allows users and system administrators to check the values of these features periodically as the system continues to learn in a culture and post-hoc after a problem has been flagged. This facilitates a better understanding of the AI’s behavior, leading to faster solutions to problems than typical black-box models based on monolithic neural networks with billions of parameters whose behavior is harder to analyze [1,63]. Assuming it can be scaled, the IRL-based approach could thus enhance AI safety through greater explainability.

### 4.1 Additional considerations and limitations

There are several limitations of our work that could be addressed in future research. First, the current study only measured behavior and did not examine humans’ beliefs or intentions toward the computer-controlled agent. Without such information, it is not possible for the current data to address issues arising in contemporary theory, such as Moral Foundations Theory, which suggests the possibility that peoples’ intent toward AI agents could be related to their perceptions of threat or affirmation from this new technology [64].

Second, our work only involved two cultural groups and a single behavior related to altruism. It would be interesting to deploy AI agents *de novo* in a wide range of cultural groups, and analyze the relationship between the learned reward functions and each cultural group’s values and behavioral tendencies. Such an approach could be used to advance basic and applied research in human-computer interaction, AI, and developmental and cultural psychology as well as in anthropology.

Third, in terms of our IRL implementation, the features used were engineered specifically for the Overcooked game environment. A topic worthy of further investigation is to endow future IRL-based AI agents with feature-learning abilities that allow them to learn features that generalize across a wide range of problems. While deep learning and transformer-based architectures offer a rich potential for learning features for IRL, the learned features may be hard to interpret, reducing interpretability. Future work could employ explainable AI techniques [59,60] for feature learning, with the goal of achieving both generalizability across tasks and interpretability.

Fourth, the results from our simplistic online game scenario may not generalize to complicated real-world situations. More broadly, it remains to be seen if IRL can be deployed in real-world and real-time scenarios, such that, if new groups of humans were to interact with an AI agent on a novel task, the AI agent can rapidly discern cultural values and use its learned reward function to interact with other humans and with other AIs in society [8]. The main questions to be answered in this regard are the amount of data needed for adapting an existing reward function or learning a new one, and what new reward features, if any, need to be introduced in the agent’s architecture or learned from human interaction.

Fifth, our online game provided participants with a single clear choice for altruistic behavior (give an onion to the other player or not) but realistic scenarios involving altruistic decision making can be more nuanced: there may be no “right answer” or clear “positive” or “negative” reward values associated with choices. Testing the proposed approach on more nuanced decision making scenarios is an important direction for future work.

Sixth, the decisions involved in the Overcooked game were not particularly high-stakes: altruistic action only hurt the participant’s performance in the game in terms of costing time, resulting in fewer soups being delivered. Although participants were paid to participate in the game (see Methods), there was no payout incentivizing cooperation or noncooperation in the game. This may have accentuated the difference between the two groups of human participants tested. It would be intriguing to further examine introducing a monetary payout based on the number of soups delivered, resulting in a monetary cost that would be incurred as a result of engaging in altruistic behavior. It may be the case that, in such a situation, the cultural difference we observed could be different than in the current study.

Finally, it would be informative for future research to test whether Latino and White participants differ in performance in the donation game, as predicted by the performance of our AI agents that were trained on the Latino versus White data in the Overcooked game. If the human data does not align with the predictions of the AI model, this implies that the AI is culturally misaligned. This could be because learning from one narrow context (such as the Overcooked task) may not fully capture the multifaceted nature of cultural values. For example, Cronk [65] reported that men from the Maasai of East Africa behave differently in the classic “Trust Game” when the game is framed according to a local gift-giving tradition. This example points to the need for training IRL-based models on a broad set of human data, contexts and tasks, and a rich and diverse set of features for capturing salient aspects of these contexts and tasks. Likewise, given such an AI model, it would be important to empirically assess the degree to which the model is reflecting the actual values held by human cultural groups. These are important issues for future research to address.

### 4.2 Broader implications and conclusion

Our findings engender a need for considerations of ethical issues involved in building safe and effective AI frameworks that can match the positive social behavior and altruism that humans show in their communities. For example, the emerging issue of AI anxiety is tied to peoples’ belief that the training of AI to embody human values and behavior will be detrimental to society [66]. Although designing AI that can be tuned based on cultural experience may ameliorate such concerns, it also opens up the possibility that the AI will learn potentially undesirable behavior and practices, including stereotypes, prejudice, and discrimination, if these are prevalent in the cultural group the AI is interacting with, as has been demonstrated in human children who acquire implicit racial biases from observing adults [67,68] and in AI systems trained on biased training sets [69,70]. In this regard, we note that our proposed approach of AI learning from human interactions does not prevent the incorporation of filters or other mechanisms that (i) monitor the inputs to the AI during learning and (ii) monitor the outputs of the AI during behavior to prevent the learning and execution of behavior that can be regarded as discriminatory, or otherwise violate the law or principles regulating the conduct of the society in which the AI is operating. Our approach could thus be augmented with methodologies to evaluate the acceptance and psychological impact of AI models [64,66].

An additional concern is the potential for an approach, such as the one proposed here, being used by bad actors to enable an AI agent to mimic a person’s cultural identity, thereby gaining the person’s trust surreptitiously. In contrast to humans, AI agents could exploit this ability at scale, potentially infiltrating and disrupting societies as a result. An important direction for future work is therefore to study when cultural mimicry is beneficial and when it could become harmful, putting appropriate safeguards in place to prevent abuse of culturally-adaptive AI technologies.

Finally, the development of powerful AI agents that can perform a range of tasks across business, education, and communication is raising a host of ethical issues [71,72]. In the present work, an AI agent learned (using IRL) from the behavior of groups of humans within the context of game play, and used what it had learned to inform its own novel interactions. In a real-world context, the emphasis on speed for commercializing new AI applications could dissuade careful, culturally-attuned AI of the kind we are proposing. Alternatively, culturally-attuned AI may actually promote user enrollment on real-world platforms, a possibility that may promote user satisfaction and commercial success.

In conclusion, our work provides a proof-of-concept demonstration of how IRL could be used to learn culturally-attuned AI by applying it to the problem of learning a limited type of altruistic behavior in an online game setting. The IRL approach is more general than our demonstration and could in principle–and with more training beyond the narrow game setup in the current study–learn complex multi-dimensional reward functions quantifying more abstract moral and ethical codes driving and constraining human behavior. Exploring how such reward functions can be learned from large-scale complex human interactions is an important direction of future research, aimed at understanding how, like a child, an AI can learn and enact the cultural patterns and value systems to which it is exposed to during its development.

## 5 Methods

### 5.1 Procedure and dataset

Our online experiments were approved by the Institutional Review Board (IRB) of the University of Washington and all experiments were performed in accordance with the institution’s relevant guidelines and regulations. The human participant recruitment period was between November 29th 2021 and September 10th of 2022. Informed consent was obtained in written form from all participants at the start of each online experiment using a checkbox before they could proceed to the experiment (no minors were recruited). Our sample of *N* = 300 participants was estimated based on a power analysis (α=0.05 and 90% power) considering the altruistic effect across participants’ self-declared ethnic background of *f* = 0.18 (Cohen *f*). This effect was calculated based on the initial sample of 100 participants. Two-thirds of our participants (*n* = 203) were recruited through the Prolific platform. Due to institutional restrictions, we recruited the rest of our participants using the Positly (*n* = 59) and the MTurk (*n* = 38) platforms. The participants’ experience with the study was exactly the same on all platforms, as they were redirected to our website when they accepted the offer. We used Prolific because of its advanced population qualifiers, which allowed a more precise pre-screening of participants who were paid US$ 2.00 for an average of 10 minutes of their time. Note that participants were paid for participation, not based on the number of soups delivered in the game (see [73]). Tables 1 and 2 present the demographic characteristics of the data we collected as the first step of our study.

**Table 1:** Demographic characteristics of the 300 participants in our dataset.

Gender	Political	Race	Age
Female: 205	Left: 141	Latino: 110	Mean: 30.6
Male: 95	Center: 101	White: 190	*SD*: 10.4
	Right: 58		Median: 28

**Table 2:** Demographic breakdown of Latino and White participants in our dataset.

Race	N	Female	Male	Mean Age	Political:	Political:	Political:
					Left	Center	Right
Latino	110	51.8%	48.2%	30.1	54.6%	20.9%	24.5%
White	190	77.9%	22.1%	30.8	42.6%	41.1%	16.3%

After responding to the demographics questionnaire, participants engaged in three training steps on the game mechanics. Participants could progress only if they demonstrated learning the basic actions of the game: move around the kitchen, move ingredients to the stove location, and deliver a soup. In fact, all participants were able to either deliver a soup or provide help at least once through the three rounds of the game, with 90% of them delivering soup or providing help at least four times and half of them doing so more than ten times.

### 5.2 Model training

We used data from the first round of the game as the training dataset for IRL. Assuming that the participant’s behavior after picking up an onion (either to give the onion to the other player or to use it to cook one’s own soup) best represents the difference between the two cultural groups, we extracted these “onion-delivering” traces for the 300 participants. A trace starts with the participant picking up the onion and ends with dropping off the onion at the ‘cooperation bridge’ or the player’s own pot. Each trace is labeled as ‘altruistic’ or ‘non-altruistic’ depending on the player’s placement of the onion. Traces were also labeled according to whether the trace is from a player who had self-reported on the demographics questionnaire as being Latino or White. We finally compacted the traces by removing states where the participant-controlled agent did not move. This resulted in a total of 2958 traces, out of which 476 were labeled as “altruistic.” The traces were used to create four different training datasets based on their labels: an altruistic dataset, a non-altruistic dataset, a Latino dataset, and a White dataset.

### 5.3 Inverse reinforcement learning (IRL)

We used the Maximum Entropy Deep Inverse Reinforcement Learning approach [74] paired with Proximal Policy Optimization (PPO) [75] as our reinforcement learning (RL) algorithm and Population Based Training (PBT) [76] for hyperparameter tuning. The reward function was implemented using a neural network architecture comprising two layers, a linear input layer to transform an input vector into a hidden vector of size 200. The Exponential Linear Unit (ELU) activation function was then applied to this hidden vector. A linear output layer then converted the hidden layer activity into an output scalar reward value.

A set of features, chosen based on heuristics (see Table 3), was extracted from each game state as the input to the reward function. These particular heuristic features were selected because they provide useful information important for solving the task. They are thus helpful in defining a reward function for the task. The features include the current location of the agent relative to important attributes of the task such as stove, bridge, etc. as well as the current state of the agent and state of the game such as whether the agent has an onion and whether there is an onion on the bridge. The 2-D features in Table 3 represent (x,y) location of the agent relative to the object in question within the game’s discrete maze environment (origin is at the top left) while the 1-D features are binary variables representing “yes”/“no” answers as 0/1 values for each feature. The agent’s orientation is 4-D because we use a 1-hot vector to represent the agent facing North, East, South and West at the current location in the maze. The feature “agent on shortest path from starting position to stove with onion in hand” is also 4-D because we use a 1-hot vector to indicate the agent’s position in one of the 4 locations along the path from the square in front of the onion store to the square in front of the stove. The overall feature vector thus has a size of 18.

**Table 3:** Features used for IRL with their vector sizes. Here, “agent” refers to the AI-controlled agent operating on the left side in the original game layout (Fig 1) and “other agent” refers to the agent on the right side. Features were chosen and fine-tuned based on heuristics and training results.

Features	Vector Size	Features	Vector Size
agent’s position relative to onion store	2	onion on bridge	1
agent’s position relative to bridge	2	onions in pot	1
agent’s position relative to stove	2	agent has onion	1
agent’s orientation	4	other agent has onion	1
agent on shortest path from starting			
position to stove with onion in hand	4		

The training objective was to learn a reward function such that the RL policy maximizing expected reward according to the learned reward function imitates the human behavior in the data used for training. Thus, we evaluated the reward function by comparing the similarity between the learned policy and human behavior (using mean squared error between state trajectories generated by the policy and humans).

To train the reward function, we used an SGD optimizer with a learning rate of 0.001 and weight decay of 0.9. We used an exponential LR scheduler with gamma = 0.999. The start state of the training is shown in Fig 4 for the different layouts. The blue agent (on the left side) starts at the position shown and needs to decide whether to take actions to share the onion by placing the onion on the bridge or to place the onion in its own pot. We trained four reward functions using the four types of behaviors generated by the altruistic agent, non-altruistic agent, Latino participants, and White participants.

**Algorithm 1:** IRL for Learning a Reward Function from Human Data

**Input:** Human demonstrations, features

**Output:** Reward function neural network weights $\theta^{*}$

1. Initialize reward function neural network weights $\theta^{1}$.

2. For n=1:N:

(a) Set reward function *r*^*n*^ to the neural network $NN(f,\theta^{n})$ where *f* is the input vector of feature values.

(b) Obtain a policy $\pi^{n}$ based on reward function *r*^*n*^ using Proximal Policy Optimization (PPO) method for reinforcement learning [75], with hyperparameter tuning based on Population-Based Training (PBT) [76].

(c) Use the policy $\pi^{n}$ to get the expected state visitation count $𝔼[\mu^{n}(s)]$ for all states *s* (see [74]).

(d) Calculate the gradient of the maximum entropy data loss $L_{D}^{n}$ with respect to reward function *r*^*n*^:

$$\frac{\partial L_{D}^{n}}{\partial r^{n}}=\mu_{D}-𝔼[\mu^{n}]$$

where $\mu_{D}$ is the expected state visitation count based on the human demonstrations (see [74] for details).

(e) Update the weights $\theta^{n}$ by backpropagating the above difference in visitation counts as an error signal through the neural network $NN(f,\theta^{n})$ to obtain the new weights $\theta^{n+1}$.

### 5.4 Generalization: The keep or donate problem

To demonstrate generalization to a new scenario involving altruism, we developed a new “Keep or Donate” problem (Fig 6). A brief description of the problem was included in the main text. We provide here a more complete description. In this problem, the AI agent needs to decide, at each time step, whether to keep or donate a fixed amount of a limited resource to another agent who is resource-poor. We assume here for concreteness that the resource is money (in arbitrary units). At each time step, both agents have unpredictable expenses resulting in a deduction of either 1 or 2 units from the current balance of each agent. An expense of 1 had a 0.8 probability compared to an expense of 2 (0.2 probability) at each time step. The AI agent gets a periodic infusion (“salary”) of 5, 10 or 15 units every 6, 8 or 10 timesteps (both the salary and timesteps were chosen uniformly at random) while the other (resource-poor) agent gets paid 2 units at the same chosen timestep. At each timestep, the AI agent needs to decide whether to keep its current balance or donate 1 unit to the other agent. Each episode, which began with the AI agent having 5 monetary units and the other agent having 2 units, lasted 100 time steps.

For this new problem, the AI agent can generalize the reward features learned using IRL from human behavior in the Overcooked game (Fig 3b) by considering “onions” as a specific example of a limited resource. This allows the agent to generalize, for example, the feature “other agent has onion” to “other agent has the resource” and the feature “onions in pot” to “I have the resource”. These two generalized features were applied by the agent to the “Keep or Donate” problem with the reward values the same as the values learned from human behavior in the Overcooked game:

Besides the IRL-learned rewards in the table above, there is a penalty of –1 whenever the current balance goes to 0, capturing the undesirability of being left resourceless.

Based on the above reward function, we used standard Q-learning to learn the policy for the AI agent. The state included X, the current balance of the AI agent, and Y, the current balance of the other agent. The features in Table 4 were computed from X and Y based on whether these values were greater than zero (i.e., whether the respective agent has “the resource”). There were two possible actions for the AI agent: “Keep current balance” or “Donate 1 unit.” The Markov dynamics of the problem is illustrated in Fig 6. The first “payday” was after 6 time steps. We ran Q-learning for 100,000 episodes, with an initial learning rate of 0.5 and an epsilon of 1 (for the epsilon-greedy action selection method). The learning rate was halved every 5000 episodes. The epsilon value decayed gradually with the number of training episodes (0.00002 was subtracted from it after each episode), with the final epsilon value capped at 0.1.

**Table 4:** Generalized features used for the “Keep or Donate” game and their reward values.

Agent	“Other agent has the resource”	“I have the resource”
Non-Altruistic	0	1
White	0.08	1
Latino	0.27	1
Fully Altruistic	1	1

## Supporting information

S1 Appendix Influence of other demographic characteristics. (PDF)

S2 Appendix Example trajectories of IRL-based AI agents. (PDF)

S3 Appendix Varying parameters in the donation game. (PDF)
