# Supplementary material for: Culturally-attuned AI: Implicit learning of altruistic cultural values through inverse reinforcement learning
Source: PLoS One. 2025 Dec 9;20(12):e0337914. doi: 10.1371/journal.pone.0337914 (PMC12688098; doi:10.1371/journal.pone.0337914)
Supplement: S1 Appendix — (PDF) [file pone.0337914.s001.pdf]

S1 Appendix. Influence of Other Demographic Characteristics.

During our analysis of altruistic behaviors in our data, in addition to the influence of cultural background (Latino versus White; see main text), we found two other demographic characteristics influencing behavior that may be worthy of investigation in future studies:

- *Gender:* While controlling for age, gender, and political orientation in the data from our online experiment, we found that male participants tended to be more altruistic (about 10% more) than female players in Round 1 (Figure S1a). Although this effect was double the size of the one identified overall when comparing the two cultural groups, we confirmed that the Latino participants as a group were significantly more collaborative than the White participants.

- *Political Orientation:* We also found a relation between experimental treatment in Round 2 and political orientation. As shown in Figure S1b, those who received help in Round 2, and who held left-leaning political views, showed more altruism in Round 3 compared to those with centrist political views. This relation was not evidenced among those who did not receive help, i.e., their altruism in Round 3 was untethered from their political ideology. A power analysis of the effect of helping behavior across the three political-leaning groups (Cohen $f = 0.14$) shows a need for larger sample size ($n = 205$ per group) to rule out any false negative errors.

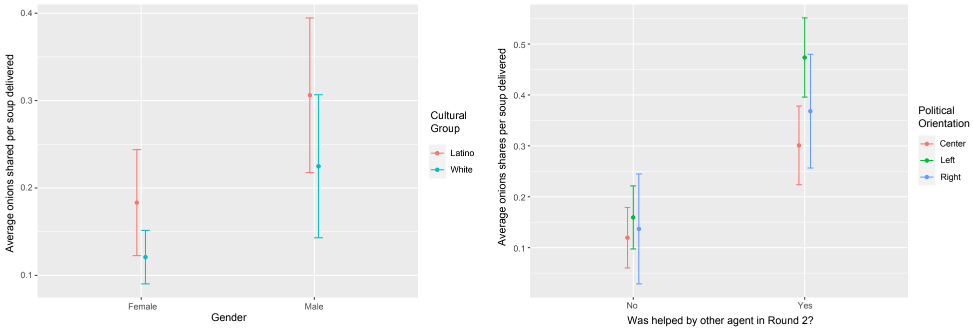

(a) **(b)**

Fig S1. Influence of Other Demographic Characteristics on Altruistic Behavior in the Online Experiment. **(a)** Influence of gender. **(b)** Influence of political orientation.
