# Supplementary material for: Culturally-attuned AI: Implicit learning of altruistic cultural values through inverse reinforcement learning
Source: PLoS One. 2025 Dec 9;20(12):e0337914. doi: 10.1371/journal.pone.0337914 (PMC12688098; doi:10.1371/journal.pone.0337914)
Supplement: S2 Appendix — (PDF) [file pone.0337914.s002.pdf]

S2 Appendix. Example Trajectories of IRL-based AI Agents

Figures S2 and S3 show snapshots from example trajectories of two AI agents trained using IRL based on data from Latino and White participants, respectively. The first trajectory illustrates an example of altruistic behavior (sharing an onion) while the second illustrates an example of non-altruistic behavior (using the onion to cook its own soup).

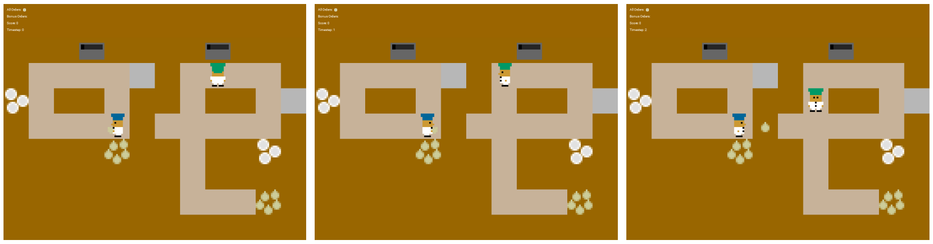

Fig S2. Example of altruistic behavior by an IRL-based AI agent trained using data from Latino participants in the online experiment. This trajectory (from the original game layout; read panels from left to right) illustrates how the trained agent (with blue hat) has learned, as part of its policy, to turn towards the cooperation bridge and provide assistance to the other agent by placing an onion on the bridge.

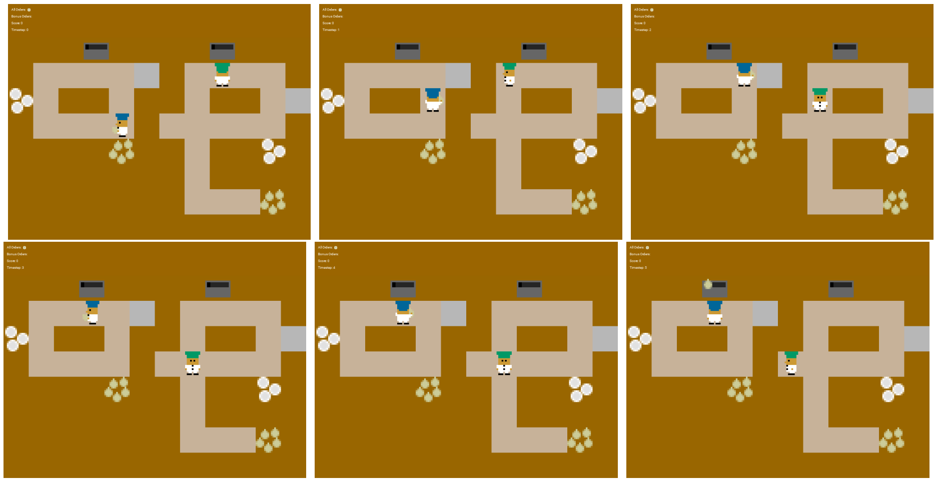

Fig S3. Example of non-altruistic behavior by an IRL-based AI agent trained using data from White participants in the online experiment. This trajectory (from the original game layout; read left to right, top to bottom) illustrates how the trained agent (with blue hat) has learned, as part of its policy, to navigate to the stove (rather than the cooperation bridge) to place the onion in the pot for cooking its own soup.
