# Supplementary material for: Culturally-attuned AI: Implicit learning of altruistic cultural values through inverse reinforcement learning
Source: PLoS One. 2025 Dec 9;20(12):e0337914. doi: 10.1371/journal.pone.0337914 (PMC12688098; doi:10.1371/journal.pone.0337914)
Supplement: S3 Appendix — (PDF) [file pone.0337914.s003.pdf]

S3 Appendix. Varying Parameters in the Donation Game

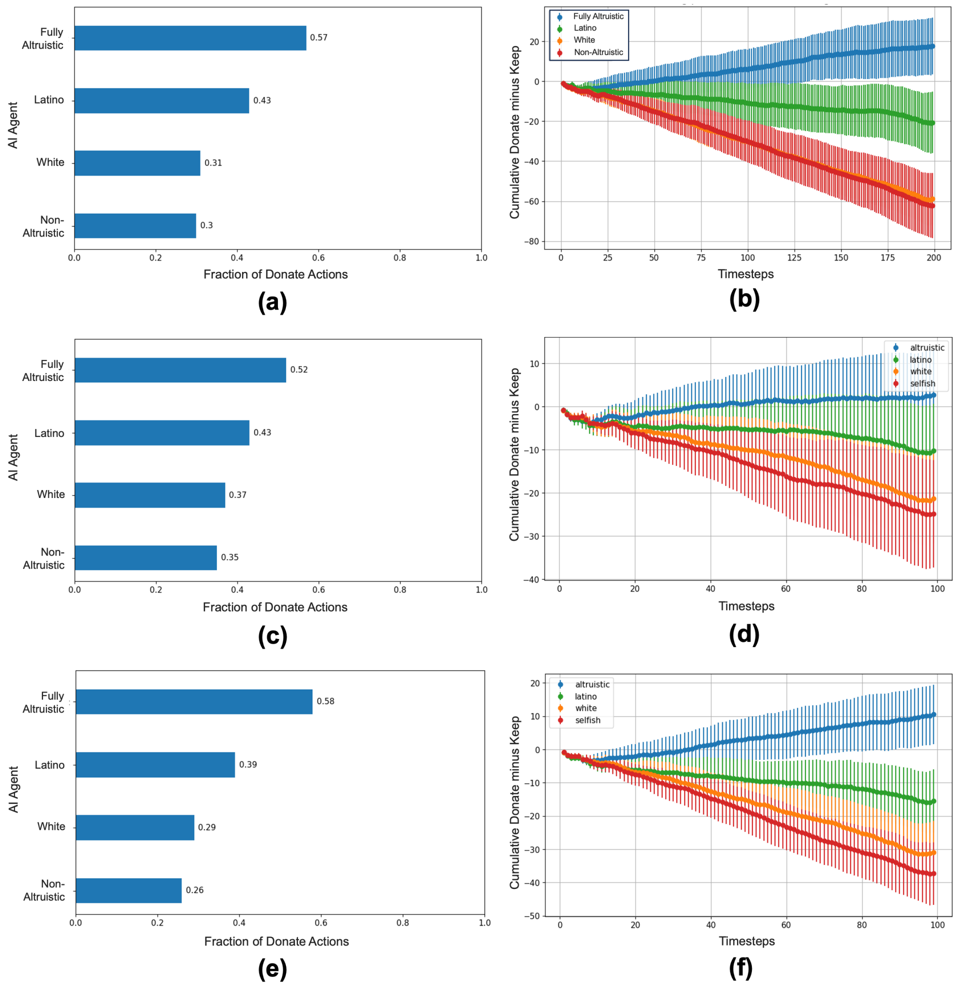

Fig S4. Effect of Varying Parameters in the Donation Game. **(a, b)** Length of each episode was changed to 200 time steps (instead of 100 time steps used for the results in the main text). **(a)** Fraction of Donate actions for each type of agent, averaged over 100 episodes. **(b)** Cumulative sum of Donate (+1) and Keep (-1) actions over the timecourse of an episode, averaged over 100 episodes (error bars show 1 standard deviation above/below mean). **(c, d)** Length of each episode was kept at 100 time steps but expense deduction probabilities were changed to 0.9 for a -1 unit expense and 0.1 for a -2 unit expense at each time step of an episode. **(e, f)** Same as (c, d) but expense deduction probabilities were changed to 0.7 for a -1 unit expense and 0.3 for a -2 unit expense. See main text for details.
